# Varied Reports of Adult Transgender Suicidality: Synthesizing and Describing the Peer-Reviewed and Gray Literature

**DOI:** 10.1089/trgh.2016.0036

**Published:** 2017-04-01

**Authors:** Noah Adams, Maaya Hitomi, Cherie Moody

**Affiliations:** ^1^ Faculty of Health Professions, School of Social Work, Dalhousie University, Halifax, Nova Scotia, Canada.; ^2^ Applied Social Psychology, College of Arts and Science, University of Saskatchewan, Saskatoon, Saskatchewan, Canada.; ^3^ Educational and Counselling Psychology, Faculty of Education, McGill University, Montreal, Quebec, Canada.

**Keywords:** transgender, suicide, marginalization

## Abstract

**Purpose:** This article reports on the findings of a meta-synthesis undertaken on published gray transgender suicidality literature, to determine the average rate of suicidal ideation and attempts in this population.

**Methods:** Studies included in this synthesis were restricted to the 42 that reported on 5 or more Canadian or U.S. adult participants, as published between 1997 and February 2016 in either gray or peer-reviewed health literature.

**Results:** Across these 42 studies an average of 55% of respondents ideated about and 29% attempted suicide in their lifetimes. Within the past year, these averages were, respectively, 51% and 11%, or 14 and 22 times that of the general public. Overall, suicidal ideation was higher among individuals of a male-to-female (MTF) than female-to-male (FTM) alignment, and lowest among those who were gender non-conforming (GNC). Conversely, attempts occurred most often among FTM individuals, then decreased for MTF individuals, followed by GNC individuals.

**Conclusion:** These findings may be useful in creating targeted interventions that take into account both the alarmingly high rate of suicidality in this population, and the relatively differential experience of FTM, MTF, and GNC individuals. Future research should examine minority stress theory and suicidality protection/resilience factors, particularly transition, on this population.

## Introduction

In recent years, transgender individuals have rapidly gained visibility. Despite this, they continue to be at risk for negative life events that adversely affect their health and well-being, such as being rendered invisible, experiencing isolation, and being subjected to societal violence.^1^ Perhaps as a result, current and past studies report appallingly high rates of suicidality (attempts and ideation) in this population. For instance, Scanlon et al.,^1^ reported an attempt rate of 43% and an ideation rate of 77% among transgender Ontarians, compared to 0.5% and 3.7%, respectively, in the general population.^2,3^ Unfortunately, the applicability of these findings in guiding policy and future research is impacted by wide variation in the findings of suicidality rates.^4,5^ For example, Xavier et al.^6^ report an attempt rate of 25% and an ideation rate of 65%, while Imbimbo et al.^7^ record rates of 3% and 50%, respectively. The current meta-synthesis attempts to address these discrepancies by measuring the overall variation across and averaging the suicidality rates within these studies. This knowledge may encourage the creation of targeted and strategic mental health interventions and research.

### Literature review

#### Historical trends

The field of transgender healthcare has been described by Bockting^8^ as evolving from utilizing a disease-based lens of transgender health and behavior to an identity-based lens. Within this framework, and as confirmed by our literature review, the disease-based lens appears to have been prevalent from 1953, when the first report on transgender suicidality was published^9^ until roughly 1997. This period was characterized by research that viewed transgender health and identity as fundamentally psychopathological, deviant, and manipulative.^10–13^ Despite no empirical support, many healthcare professionals tended to view suicidality as a manipulative tactic to force surgery,^14^ which would invariably lead to poor mental health outcomes, such as suicide and psychotic breakdown.^15^ In fact, evidence existed that suggested the opposite: that improvement tended to be drastic following surgery.^16^

The utilization of an identity-based lens appears to have begun to overtake the disease-based lens in roughly 1997, with the publication of Devor's^17^ seminal study on female-to-male (FTM) transsexuals. The identity-based lens is characterized by the assumption that transgender identity is part of a natural spectrum that includes identities beyond those that are male or female, feminine or masculine.^8^ Proponents of this lens argue that physical and psychological stress, resultant from institutional and societal discrimination, are major contributors to suicidality in this population.^1,18,19^

While the disease-based lens continues to be used,^8^ particularly outside of North America,^20^ there is increasing disagreement as to whether the cause of transgender identity is mental, hormonal, biological, and/or genetic.^21^ Similarly, suicidality in this population is variously attributed to psychopathology, neurobiology,^22^ and/or antitransgender stigma.^19^

#### Meta-analyses and systematic reviews

Ten systematic reviews or meta-analyses have mentioned suicidality among transgender people; however, only Marshall et al.,^23^ approached this as the primary question and was designed to include all articles on transgender suicidality. Nevertheless, although an important contribution to the literature and even accounting for the parameters of their review, Marshall et al.^23^ appear to have missed several studies (numbers 2, 5–11, 16, 19–21, 23–26, 32–33, 35, and 42 in Table 1), and counted a single research project three times (37 in Table 1).

**Table 1.** **Studies Included in the Meta-Synthesis**

	Author/s	Population	Time measure	Ideation	Attempt	Description
1	Cole et al.^24^	318 MTF 117 FTM	Before/after treatment		Before treatment All: 15% MTF: 12% FTM: 21%	Analyzed the incidence of Axis 1 and 2 diagnoses among patients at a Texas gender clinic from 1980 to ∼1997. Patient charts included information garnered from a clinical interview and questionnaire administered on first contact. Psychometric inventories also utilized in some cases.
					After treatment All: 0%	
2	Devor^17,25^	45 FTM	Lifetime	FTM: 28.89%	FTM: 22.22%	Investigated the life experiences of FTMs via the first needs assessment conducted on the subject of transgender suicidality. Data collected from 1988 to 1992. Suicidality information was volunteered by all participants during the interview.
3	Rehman et al.^26^	28 MTF	After surgery	MTF: 7.14%		Investigated postsexual reassignment sex and surgery satisfaction in MTF patients of a NYC hospital from 1980 to 1994. Participants were asked whether they experienced suicidal ideation before and/or after surgery. Only postsurgical information is reported.
4	Mathy^27^	21 FAAB 49 MAAB 3 Intersex	Lifetime	All: 37%	TG: 23.30%	Examined suicidality among transgender respondents to two large surveys of human sexuality conducted on the MSNBC website over 1 month in 2000. First survey was a selected random sample and the second invited every thousandth visitor to participate. Transgender respondents were forced to select male, female, or transgender and represented 0.2% of each sample. Transgender participants were compared to 1083 heterosexual females, 1077 heterosexual males, 73 psychosocially matched cisfemales, 73 psychosocially matched cismales, 256 homosexual females, and 356 homosexual males.
5	Singer et al.^28^; Kenagy^29^; Kenagy^30^	23 FTM 38 MTF 25 GNC 17 CD	Lifetime		All: 38.83% MTF: 52.63% FTM: 43.48% TG: 28% CD: 17.65%	Employed a needs assessment to explore physical and mental health among Philadelphia-area transgender individuals from 1996 to 1997.
6	Kenagy and Bostwick^31^	33 FTM 78 MTF	Lifetime	All: 61.69%	All: 26.27%	Employed a needs assessment to explore the health and social service needs of transgender individual's in Chicago over 6 months (2000–2001) via snowball sampling and using trained transgender interviewers.
7	Bockting et al.^32,33^	141 MTF 34 FTM	In last 3 years	All: 52%		Sought to analyze the impact of an 8-week sexual health seminar (offered from 1997 to 2002) in Minnesota on health-risk factors, such as HIV risk behavior in a group of LGBT individuals. Transgender participants were compared to 480 “men who have sex with men” and 122 “women who have sex with women.”
8	Kenagy^29,30,34^	49 MTF 32 FTM	Lifetime	All: 46.91% MTF: 59.18% FTM: 28.13%	All: 19.75% MTF: 28.57% FTM: 6.25%	Employed a needs assessment to investigate the health and social service needs of a Philadelphia-area transgender community over 6 months in 1997.
9	Risser et al.^35^	63 MTF 4 cismales	Attempts over lifetime	Lifetime All: 58.21%	Lifetime All: 29.85%	Employed a needs assessment to investigate the social and sexual health status of a group of transgender women in Houston over 2 months (2002–2003).
			Ideation over lifetime and in past 30 days	Past 30 days All: 16.42%		
10	Xavier^36^; Xavier and Simmons^37^; Xavier et al.^38^	7 FTM 12 MTF 183 GNC 4 intersex 23 ciswomen 23 cismen	Lifetime	All: 34.92%	All: 16.27%	Employed a needs assessment to investigate the health and social service needs of a transgender community in Washington, DC over 4 months (1999–2000). The number of respondents is amalgamated from the slightly different numbers published in the three articles.
11	Clements-Nolle et al.^18^; Clements-Nolle et al.^39^	123 FTM 392 MTF	Lifetime		All: 32.23% MTF: 32.40% FTM: 31.71%	Examined HIV, risk behaviors, mental health and healthcare use of transgender individuals in San Francisco over 5 months in 1997.
12	Zians^40^	16 FTM 57 MTF 56 TG	Ideation past 12 months	Past 12 months All: 31.62%	Lifetime All: 17.65%	Employed a needs assessment to investigate the healthcare and social service needs of transgender individuals in San Diego over 7 months in 2004. The manner of inquiry into suicidality was somewhat unclear.
			Attempts over lifetime			
13	Taylor^41,42^	21 FAAB 49 MAAB 3 intersex	Lifetime	All: 54%	All: 28%	Employed a needs assessment to investigate the health and social service needs of transgender and two-spirit individuals in Manitoba and Northwestern Ontario over 6 months in 2006. Contains an unusually high rate of Aboriginal participants (27.40%). Participants were given the option to complete either a long or short form questionnaire where the short form did not inquire into suicidality. Suicidality figures presumed to be for all 73 respondents, regardless as to whether they originate from the long or short survey.
14	Landers and Gilsanz^43^	52 TG	Last 12 months	All: 30.77%		Conducted for the Massachusetts Department of Public Health over 10 days in 2009, using the e-mail list of MassEquality, in part to determine the impact of Massachusetts' equal marriage law on LGBT health and security. The overall response rate was only 4.2% and transgender respondents were forced to select LGB or transgender. Transgender participants were compared to 450 heterosexual, 965 gay/lesbian, and 136 bisexual individuals.
15	McDuffie and Brown^44^	4 FTM 55 MTF 11 GNC	Lifetime	All: 60.71%	All: 10.71%	Analyzed chart data for U.S. Armed Forces Veterans examined for gender identity disturbances at a Tennessee Veterans Affairs office from 1987 to 2007. Likely overlap with Blosnich et al.^62^ and Brown and Jones.^84^
16	Nuttbrock et al.^19^	571 MTF	Lifetime	MTF: 53.50%	MTF: 27.90%	Sought to determine the psychiatric impact of gender-related abuse across the life course of transwomen via a large-scale longitudinal study, conducted in New York City, from 2004 to 2009. Obtained responses for up to five distinct time periods (early adolescence, late adolescence, early adult, young adult, early middle age). Suicidality information elicited from respondents positive for either of the depression screens at any of these periods, with responses to suicidality questions scored as 1 (yes) or 0 (no) and the total for all three questions added to form a suicidality score.
17	Nemoto et al.^45^; Operario and Nemoto^46^; Nemoto et al.^47^	573 MTF	Lifetime	MTF: 54.97%	MTF: 33.33%	Undertaken over 8 months from 2000 to 2001 and again from 2004 to 2006. Recruited primarily San Franciscan transgender women of color (African American, Latina, Asian/Pacific Islander) with histories of sex work. Primarily sought to gauge the impact of HIV, but also investigated socioeconomic status, victimization, physical and mental health.
18	Maguen and Shipherd^48^; Shipherd et al.^49^	22 FTM 60 MTF 32 GNC 28 CD	Lifetime		All: 18.31% MTF: 20% FTM: 40.91% GNC: 9.38% CD: 7.14%	Examined suicidality among participants at a transgender conference in New England known to focus on CDs and may have under sampled FTM, MTF, and GNC individuals.
19	Effrig et al.^50^; Hayes et al.^51^	97 TG	Lifetime	All: 53.61%	All: 27.84%	Discusses victimization and psychological distress among transgender college students via clinical (Fall 2008) and nonclinical (Spring 2010) samples. The clinical sample is comprised of college counseling center patients where, unlike the nonclinical sample, “other” was not a gender option. The nonclinical sample consisted of respondents to a survey conducted by colleges aligned with the counseling centers. Both used the same survey measure; however, institutions varied in their use of incentives and completion prompts. Effrig et al.^50^ contains contradictory figures for both the number and suicidality of nonclinical and clinical participants, although it was possible to rationalize these figures, revealing a probable 65 nonclinical and 32 clinical participants. We have combined the nonclinical and clinical data because the articles don't provide enough raw data to determine each samples' individual attempt rates.
20	Meier et al.^52^	367 FTM	Lifetime		FTM: 43%	Investigated the effect of gender confirming hormonal treatment on FTM individuals over 3 months in 2008.
21	House et al.^53^	29 FAAB 135 MAAB	Lifetime		All: 34.8%	Explored the social and psychological experiences of sexual minorities via an internet survey of LGBT respondents conducted over 1 month in 2004. U.S. transgender respondents represented 14.6% of all participants, with their findings compared to the lesbian, gay, and bisexual respondents. Transgender participants were compared to 524 male-identified, and 438 female-identified individuals.
22	Fredriksen-Goldsen et al.^54^	46 FTM 105 MTF 23 GNC	Lifetime	All: 71.1%		Sought to investigate the health of older LGBT adults in the United States (50–90 years old) over 5 months in 2010. Transgender respondents represented 7% of all respondents, with their responses compared to LGB counterparts. Transgender participants were compared to 1462 gay men, 773 lesbians, and 127 bisexuals.
23	Xavier et al.^6^; Goldblum et al.^55^; Testa et al.^56^	121 FTM 229 MTF	Lifetime	All: 63.71% MTF: 58% FTM: 79%	All: 25.43% MTF: 41% FTM: 79%	Explored the health and service needs of transgender Virginians over 10 months (2005–2006).
24	Beemyn and Rankin^57^; Testa et al.^58^	653 FTM 2178 MTF 256 GNC	First felt transgender	All: 16.62% MTF: 16.80% FTM: 17.46% GNC: 12.89%		Explored the life experiences of transgender individuals over 3 months (2005–2006). Among other topics, it investigated whether suicidality was negatively correlated with having been exposed to positive representations of transgender individuals.
25	Heinz and MacFarlane^59^	23 FTM 21 MTF 10 GNC	Lifetime	All: 35.19% MTF: 47.62% FTM: 26.09% GNC: 30.00%	All: 27.78% MTF: 19.05% FTM: 39.13% GNC: 20%	Explored the health and social service needs of transgender respondents on Vancouver Island, British Columbia, from 2010 to 2011.
26	Brown et al.^60^	9 MTF	Before transition	MTF: 55.56%	MTF: 11.11%	Investigated the life experiences of transfeminine individuals in the Missouri-Kansas City area over an unspecified time period. All respondents spontaneously shared experiences of suicidality within the context of being pretransition.
27	Moody and Smith^61^	56 FTM 59 MTF 15 GNC 3 intersex	Attempts over lifetime Ideation over lifetime and in past year	Lifetime All: 65.41% Past year All: 74.44%	Lifetime All: 26.32%	Explored suicidality and resilience among transgender respondents in Canada, the majority of who were from Quebec and Ontario.
28	Blosnich et al.^62^	1326 TG	2011	All: 5.13%		Tracked suicidality among transgender veterans through an analysis of the U.S. Department of Veterans Affairs' electronic health records of patients with a listed ICD-9 diagnosis of “gender identity disorder.” The records searched stretch from 2000 to 2011, while records for suicidality (referred to as “suicide related-behaviors” or “events”) were only available from 2009 to 2011. Quite a bit of potential overlap with McDuffie and Brown^44^ and Brown and Jones^84^
29	Haas et al.^63^; Grant et al^64,65^	1776 FTM 3005 MTF 766 GNC 894 CD	Lifetime		All: 40.04% MTF: 41.63% FTM: 46.28% GNC: 35.64% CD: 25.84%	Examined the health and social service needs of the U.S. transgender population from 2008 to 2009 via a needs assessment. Data were partially collected from survey party, which may have increased the participation of particularly hard to find populations (e.g., homeless, or with a low literacy level).
30	Mereish et al.^66^	16 TG	Lifetime	All: 68.75%	All: 31.25%	Explored relationships between LGBT-based victimization, substance use problems, and suicidality among patients waiting for an appointment at a New England community health center from 2001 to 2003. Participants restricted to identifying their gender as either male, female, or transgender, with only 1.10% identifying as both “transgender” and a “sexual minority.” Transgender participants were compared to 1130 sexual minority men and 305 sexual minority women.
31	Reisner et al.^67^	31 TG	Lifetime	All: 58.06%	All: 29.03%	Presented research into transgender health disparities among patients waiting for a medical appointment at a Massachusetts community health center (unrelated to Mereish et al.) over the span of one year (2001–2002). Respondents asked to indicate their gender identity as male, female, or transgender and the resulting 31 transgender respondents paired with cisgender controls, matched for age (within 3 years), ethnicity, education, and income. Transgender participants were compared to two cisfemales and two cismales each. Likely some crossover with Reisner et al.^85^
32	Reisner et al.^68^	23 FTM	Lifetime		FTM: 21.74%	Examined suicidality in a cohort of FTM patients screened for STDs from July to December 2007 at a Boston community health center. Data obtained via a retrospective chart review and past suicide attempts documented in the electronic medical record. Likely some crossover with Reisner et al.^85^
33	Wilson et al.^69^; Santos et al.^70^	314 MTF	Lifetime	MTF: 52.97%		Investigated access to transition-related healthcare, as well as other physical and mental health services among transgender women in San Francisco over 4 months in 2010.
34	Rosser et al.^71^; Perez-Brumer et al.^72^	532 FTM 697 MTF	Lifetime		Lifetime All: 28.89% MTF: 23.82% FTM: 35.53%	Reported on the impact of individual and structural suicidality risk factors, specific to transgender individuals. Also investigated the social demographics of hidden sexual minorities as part a larger investigation into gender and HIV risk.
			Past 12 months		Past 12 months All: 4.15%	
35	Scanlon et al.^1^; Rotondi et al.^73,74^; Bauer et al.^75,76^; Scheim and Bauer^77^; Bauer et al.^78^	227 FTM 205 MTF 7% GNC 9% CD	Lifetime Past 12 months	Lifetime All: 77% Past 12 months All: 36% MTF: 35% FTM: 38% GNC: 31%	Lifetime All: 43% Past 12 months All: 10% MTF: 10% FTM: 11% GNC: 6%	Investigated the health and social service needs of transgender people in Ontario from 2009 to 2010.
36	Edelman et al.^79^	182 FTM 307 MTF 32 GNC	Attempts over lifetime and in past year	Lifetime All: 60% MTF: 58% FTM: 66%	Lifetime All 34% MTF 36% FTM 31%	Washington, DC transgender needs assessment conducted from May 2012–2013. Update of a prior study (No. 10). It was not possible to determine the exact number of MTF and FTM (described as transfeminine and transmasculine) respondents that experienced suicidality. As a result the suicidality figures are recorded here as reported. Similarly, there was little suicidality data for the 32 respondents that did not identify as transmasculine or transfeminine and they are, therefore, excluded.
			Ideation over lifetime		Past 12 months All 10% MTF: 14% FTM: 4%	
37	Mustanksi et al.^80^; Liu and Mustanski^81^; Mustanski and Liu^82^; Birkett et al.^83^	8 FTM 13 MTF	Attempts over lifetime and in last 12 months		Lifetime All: 52.4% Last 12 months All: 19.0%	Explored suicidality among Chicago LGBT youth from 2007 to 2008. Transgender participants were compared to 107 cismales, 119 cisfemales.
38	Brown and Jones^84^	1578 FAAB 3557 MAAB	Suicidality recorded in VA records (treated as lifetime)	All: 19.36%		Reports on mental and medical health disparities among transgender veterans receiving healthcare through the U.S. Veterans Administration from 1996 to 2013. Participants were identified by noting whether their sex marker had been changed since the time of VA enrolment. Suicidality determined from a diagnostic code in the patients file. Quite a bit of potential overlap with McDuffie and Brown^44^ and Blosnich et al.^62^ Transgender participants were compared to 15405 cisgender individuals.
39	Reisner et al.^85^	106 FTM 74 MTF	Lifetime	All: 31.11% MTF: 32.43% FTM: 30.19%	All: 17.22% MTF: 20.27% FTM: 15.09%	Assessed mental health information in electronic patient records of transgender youth at a Boston-area community health center from 2002 to 2011. Respondents were divided into FTM or MTF and matched to cisgender controls within 3 months of first being noted as transgender in their patient file and according to gender identity, age, and race/ethnicity (some partially matched for age and gender identity). Transgender participants were compared to 180 cisgender individuals. Likely some crossover with Reisner et al.^67^ and Reisner et al.^68^
40	Olson et al.^86^; Olson^87^	49 FTM 47 MTF	Lifetime	All: 51.04% MTF: 42.55% FTM: 59.18%	All: 30.21% MTF: 27.66% FTM: 32.65%	Cohort study that investigated the physiological and psychological health of transgender youth between 12 and 24 years of age (mean 19.2) who presented for gender services at a children's hospital in Los Angeles. Psychosocial health assessed via a computer-assisted self-administered survey and a selection of psychometric items, while physiologic health assessed via patient files. Only patients that presented for care from February 2011 to June 2013 were eligible.
41	Kuper^88^	1562 FAAB 439 MAAB	Lifetime	Lifetime All: 96.5%	Lifetime All: 32.30%	Reports on suicidality and gender development among a cross-sectional cohort of GNC youth and young adults from throughout the United States.
			Past year	Past year All: 80.2%	Past year All: 10.4%	
42	Irwin et al.^89^; Su et al.^90^	91 TG	Lifetime		All: 35.16%	Assessed factors leading to suicidal ideation and health disparities among LGBT Nebraskans in 2010. About 11.9% identified as transgender, where the options were male, female, and/or transgender. Transgender participants were compared to 676 cisgender LGB individuals.

CD, cross-dresser; FAAB, female assigned at birth; FTM, female-to-male; GNC, gender non-conforming; MAAB, male assigned at birth; MTF, male-to-female; TG, transgender.

The remaining nine reviews and meta-analyses, while important, largely address transgender suicidality as a secondary question in relation to other issues, such as HIV or geographical location. As such, these articles do not encompass the breadth of transgender suicidality literature addressed in this meta-synthesis. For example Meads et al.^91^ restricts itself to one region in England, while Herbst et al.^92^ focuses primarily on HIV prevalence and risk factors which, though common in this field, is not reflective of all transgender individuals.^21^ Finally, the articles by Pauly,^93–96^ the Wessex Institute for Health Research and Development,^97^ and Lundström et al.^98^ address transgender suicidality vis-a-vis the impact of gender confirmation surgery, finding the persistently held notion that transgender individuals experience higher rates of suicidality after gender confirming surgery to be unfounded (indeed, suicidality has been recorded to be most common when requests for surgery are refused).^15^

## Method

### Eligibility criteria

All studies included in this meta-synthesis reported data from original participant research investigating transgender suicide attempts and/or ideation. Both gray and peer-reviewed academic literature were included, as the former allowed for a larger data pool and helped to reduce the impact of publication bias, where research with nonsignificant findings may be published in a thesis, but not a peer-reviewed journal. Studies were included if they were published in English-language North American (Canada and the United States) journals, between 1997 and February 1, 2016. These studies must also have reported quantitative data on suicidality among five or more transgender participants with a diagnosis of gender dysphoria and/or self-identification as transgender. Further, the majority (50% +1) of participants must have been 18 years or older at the time of participation.

Additionally, a word about the inclusion of cross-dressers (CDs). It is noted that there are many terms used by transgender individuals to define their experience of gender and gender identity.^65,99^ The term CD, which identifies individuals “who dress in clothing or express their gender in ways that society deems inconsistent with the sex they were assigned at birth” is commonly used for this purpose.^99^ In the case of this study, CDs are included because they answered research calls for transgender people and because in most cases this identity cannot be assumed to be exclusive of other transgender identities.

Studies were excluded if they reported only on completed suicides or nonsuicidal self-injury, single case reports, or included a transgender cohort as part of a larger population (e.g., LGB) without providing discrete transgender data. This includes reports that referenced, but did not provide data on investigations into transgender suicidality. Additionally, research that resulted in multiple publications was only counted once, while the most recently published report was of primary import, though all reports published on a single study were reviewed for pertinent information. Finally, all included studies were reported in a primarily written format, thus excluding posters and other visual presentations.

### Data collection

Rates of transgender suicidality were collected from included studies. Data absent from the included articles were supplemented by interviews with the study authors. However, to ensure the confidentiality promised as a condition of these interviews and required by the original Research Ethics Board approval, obtained through Dalhousie University, we have not indicated where this supplementation occurred.

#### Literature

Transgender suicidality literature was primarily identified via the WorldCat database because it is one of the largest global digital catalogs of cross-referenced and multi-disciplinary material. This search was supplemented with and cross-checked against results from Google Scholar and Google Search and later by manually reviewing the reference lists of identified articles, which resulted in the identification of a small number of additional articles. University of Calgary suicidality research Richard Ramsay's collated list of transgender suicidality studies was also invaluable.^100^ The following key terms were used when searching WorldCat, Google Scholar, and in Google Search; “transgender suicide”; “transsexual suicide”; “FTM suicide”; “MTF suicide”; “transsexual suicidality”; “transgender suicidality”; “transgender suicide attempt”; and “transsexual suicide attempt.”

**FIG. 1.**
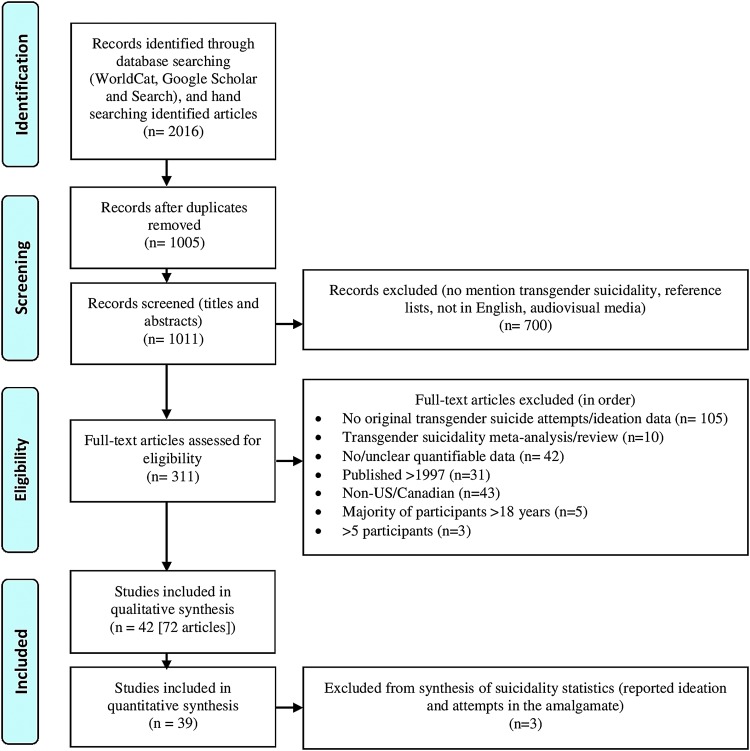
A PRISMA^101^ flow chart showing the literature reviewed for this assessment of transgender suicidality and the process of narrowing its scope to those North American studies that reported original transgender suicidality data, were published since 1997, and included five or more participants who were majority 18 years of age or older (50% +1).

Of the 2016 records identified, 1005 duplicates were immediately eliminated. The titles and abstracts of the remaining articles were screened (Fig. 1). After excluding reference lists, non-English language publications, audiovisual reports, and those that did not mention transgender suicidality in either the title or the abstract, 311 articles remained. The full texts of these articles were reviewed. Those further eliminated include articles that reported no original data on transgender suicide attempts and/or ideation (105), meta-analyses/reviews of transgender suicidality (10), those with no/unclear quantitative data (42), articles published before 1997 (31), those not from the United States or Canada (43), those where the majority of participants were under 18 at the time of first data collection (5), and those with less than five participants (3). The remaining 72 articles, representing 42 distinct research studies (some published multiple times), were examined in the qualitative synthesis, while the 3 that reported suicidality as an amalgamate of ideation and attempts were excluded from the quantitative synthesis.^32–33,62,84^

### Data analysis

The 42 studies on transgender suicidality are subjected here to a simple descriptive analysis, with the intention of summarizing the statistics on transgender suicidality included in the source research, and providing inroads to a more thorough examination in the future. To this end, individual studies' suicidality statistics (total number of participants, mean suicidality, range) were recalculated where possible using *all study participants* as a denominator. We considered but chose not to use *only participants that responded to a given suicidality question* because relatively few studies reported enough raw data to reliably and consistently calculate this figure. Similarly, we noted that the 42 studies differed in whether they reported ideation and attempts separately or in an amalgamate. We have compensated for this by separating these statistics where enough raw data are available to make this possible. Where this was not possible (three cases) we recorded the given figures as is and did not use them in subsequent calculations of cross-study suicidality averages. Note that, beyond these actions and the criteria set for inclusion on this meta-synthesis, no additional measures were taken to account for varying study designs.

After processing the individual studies as described, we combined their suicidality statistics and calculated the overall mean rate of suicidal ideation and attempts for the 39 that measured these as independent factors and excluding the 3 that combined these figures. Variation is operationalized in the current meta-synthesis as any difference between the calculated study ideation and attempt means and the overall ideation and attempt means for all studies included. After calculating these studies by comparing these means according to participants' gender identity and over time (*ever*, *past year*, and *before treatment/transition/when participants first felt transgender*). We also compared the range of suicidality figures reported by these studies. These calculations were conducted with the aid of Excel and SPSS v. 23.

## Results

### Methodologies and study design

Of the 42 studies analyzed here, 11.9% (*n*=5) were Canadian, with the remaining 88.1% (*n*=37) originating in the United States. Beginning with an average of 1 per year, there are now ∼4 publications per year on this topic, with a high of 11 in 2011. Interestingly, despite the inclusion of gray literature, only six studies were solely published outside of a formal peer-reviewed format. Of these, one took the form of a thesis, while the remaining five were published in reports to communities, research granting agencies, governmental bodies, or as part of rigorous analyses by academic think tanks.

Eleven studies have included cisgender comparison or control groups, all of which were published since 2002, and 82% (*n*=9) since 2009. In comparing the research designs of all 42 studies, we see that 11.9% (*n*=5) used case series, 59.5% (*n*=25) used cross-sectional, 19% used case control (*n*=8), and 9.5% used cohort (*n*=4) designs, with a marked increase in the latter two since 2009 (83% of 12 total). Additionally, 73.8% (*n*=31) of these studies took place in a nonclinical setting, 23.8% (*n*=10) in a clinical setting, and 2.4% (*n*=1) in combined clinical and nonclinical settings. Finally, four different methods of data collection were employed, with 69% (*n*=29) using self-administered questionnaires, 31% (*n*=13) administered questionnaires, 14.3% (*n*=6) face-to-face interviews, and 16.7% (*n*=7) chart reviews.

### Participant demographics

The individual studies that make up this meta-synthesis include from 9 to 6441 participants, for an estimated total of 25,735 transgender individuals. Of these, ∼4625 could be distinctly classified as FTM, 9698 as male-to-female (MTF), 1377 as gender non-conforming (GNC), and 939 as CDs. The majority of participants were male assigned at birth (MAAB; *n*=15,074 vs. female assigned at birth [FAAB]; *n*=8697). Additionally, though inconsistently recorded and difficult to track, four studies reported on a total of 13 intersex participants (Table 1).

The majority of studies excluded all participants under 18 years of age (*n*=32), though^36–38,40,80–83,88^ reported a minority under this age (2.94–33.3%). In the remaining cases,^1,28–30,43,50,51,73–78,85–87^ while it was apparent that the majority of the sample was over 18 years at the time of participation, insufficient information was provided to determine the exact number and proportion of those under this age.

### Suicidality

Of the 42 studies analyzed, 83% (*n*=35) asked participants about lifetime ideation and/or attempts, while 36% (*n*=15) measured suicidality specific to a particular period either instead of, or in addition to this. (In one case, suicidality was recorded over a period of 17 years, which was treated as lifetime suicidality in the following calculations.) In addition to different studies assessing either ideation and attempts during different time frames (e.g., lifetime or part year), several studies assessed both ideation and attempts over different periods (e.g., lifetime ideation, attempts in past year) and/or for different gender identities and/or sexes assigned at birth.

Suicidal ideation and attempts were typically reported separately; for example, 67% (*n*=28) of the studies assessed ideation and 81% (*n*=34) assessed attempts. Additionally, three studies (7, 28, and 38 in Table 1) amalgamated ideation and attempts into a single figure, one of which investigated suicidality over the lifetime, one in the past 3 years, and one in 2011 alone. These three studies have therefore been excluded from the subsequent synthesis of suicidality statistics.

#### Lifetime suicidality

**FIG. 2.**
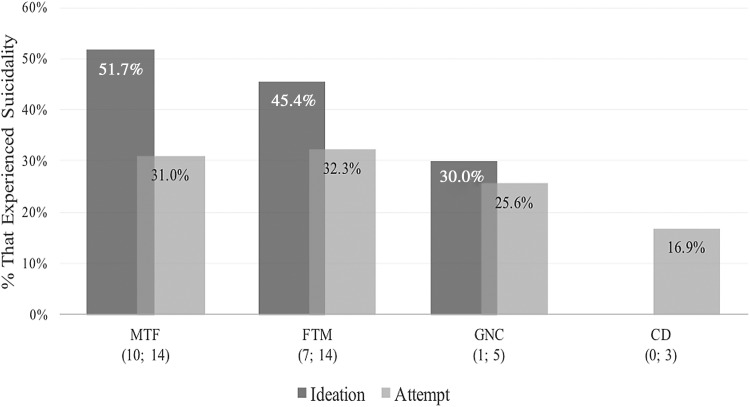
Suicidality among transgender adults in studies that measured this across the lifetime, or “ever,” as compared by gender identities. The numbers in brackets represent, first, the number of studies that recorded ideation statistics and second, the number that recorded attempt statistics. The label “All” indicates the figure for suicidality in studies that measured this among all participants, irregardless of gender identity. CD, cross-dresser; FTM, female-to-male; GNC, gender non-conforming; MTF, male-to-female.

As seen in Figure 2, when suicidality statistics are averaged across all studies and analyzed by participants' gender identity, there is a tendency for MTF participants to ideate somewhat more often than FTM participants (51.7% vs. 45.4%), while attempts are virtually identical (31% vs. 32.3%). GNC individuals appear to ideate (30%) and attempt (25.6%) less than MTF and FTM individuals, while CD individuals experience perhaps the least suicidality of the four groups (16.9% ideation).

#### Suicidality over a specific period

**FIG. 3.**
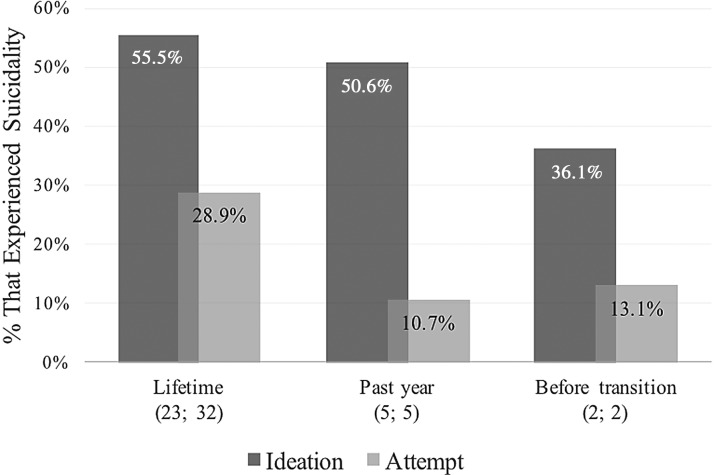
Suicidality among transgender adults in studies that measured this in the past year, compared with those that measured this before transition. The numbers in brackets represent, first, the number of studies that recorded ideation statistics and second, the number that recorded attempt statistics. Studies that measured suicidality in “the past year” and “past 12 months” were combined. Little information was available for suicidality before transition (three studies total, two each for ideation and attempts) and the category is a combination of *before treatment*, *when participants first felt trans*, and *before transition*.

As seen in Figure 3, the most common time period used for measuring suicidality was over the *lifetime* (*n*=23 for ideation; *n*=32 for attempts), followed by the *past year* (*n*=5 for ideation; *n*=5 for attempts). Additionally, four studies measured suicidality surrounding transition (e.g., when participants *first felt transgender*, *before treatment*, *before transition*, and *after surgery*). Of this group, only the three (*n*=2 for ideation; *n*=2 for attempts) that investigated suicidality *before treatment*, *transition*, or when participants *first felt transgender* (representing a combined total of 3531 individuals) could reasonably be compared. Finally, one study measured ideation in the past 30 days.

The overall *lifetime* rate of suicidal ideation (*M*=55.5%; range=28.9–96.5%) and attempts (*M*=28.9%; range=10.7–52.4%) were calculated across the 23 and 32 studies for which these behaviors were, respectively, recorded. Similarly, we see the average and range of suicidality over the *past year* (ideation *M*=50.6%; range=30.8–80.2%; attempt *M*=10.7%; range=4.2–19%). Of equal import, this data set demonstrates the average and range of suicidality *before transition* (ideation *M*=36.1%; range=16.6–55.6%; attempt *M*=13.1%; range=11.1–15%).

## Discussion

### Findings

Methodologically speaking, much has changed within the period covered by this meta-synthesis. For example, there appears to have been an increase in methodological rigor, particularly in terms of study design and the use of comparison groups. Furthermore, the majority of transgender suicidality research now takes place in nonclinical environments, which is a marked contrast from the beginning of this period and the previous era. Demographically, these studies have included proportionately more MTF and MAAB participants than FTM/FAAB, GNC, or CD. Interestingly, CD appears to be something of a remnant of an older period in North American transgender culture, no longer in widespread use, at least as seen in recent academic publications.

In the studies examined in the current meta-synthesis, an average of 56% of participants experienced lifetime suicidal ideation and 29% had *ever* attempted suicide. Given the rate of suicidality in the general population, 0.5%^2^ and 3.7%,^3^ respectively, it is clear that transgender individuals are disproportionately impacted by this phenomenon. Indeed, even over *the past year*, where 51% of participants ideated and 11% attempted, suicidality is approximately 14 and 22 times higher than in the general public. MTF participants also appear to experience more ideation and roughly equal attempts as their FTM counterparts, though both groups ideate and attempt more than participants identified as GNC, followed by those identified as CD. Finally, the vast majority of studies explored suicidality at any point in a participants' lifetime, either in addition to, or instead of time-specific measures (*past year*, *before transition*). Of those that did record time-specific measures, the majority did so for the past year.

We can also theorize on the impact of time and transition on suicidality, though only in an exploratory fashion. For example, as expected, when we compare *lifetime* to *past year* suicidality we find that ideation (55% vs. 51%) and especially attempts (29% vs. 11%) decrease. It seems counterintuitive, on the other hand, that suicide attempts are lower *before transition* (ideation 36.1%; attempt 13.1%) than over most other periods (past year attempts being the exception). However, we may expect to find higher rates of suicidality over the *lifetime* because this is a category that represents a non-transition specific and cumulative experience, in contrast to the single period represented by *before transition*. It may also be the case that individuals who do not desire transition represent a less dysphoric group and are therefore less suicidal.^64^ By contrast, evidence is mounting that barriers to transition-related healthcare contribute to suicidality among those who desire such measures^63,78^ and though it sometimes increases during transition,^78^ it typically decreases once desired transitional goals are completed.^30,102^ Indeed, a recent qualitative inquiry into suicide protective factors among trans adults identified several important protective factors among this population, one of which was socially and/or medically transitioning (for those who seek it).^103^ Additionally, suicidality may be generally higher among transgender individuals than the general population throughout the life course, due to factors unrelated to transition, such as stigma and discrimination.^20,55,78^

### Implications

The results of the current meta-synthesis can inform a number of potential transgender healthcare strategies. For example, as noted above, there is growing evidence for the role of both antitransgender discrimination and transitional services in suicidality among this population, with the former implicated in heightened suicidality and the provision of the latter in its reduction. Physical healthcare providers may, therefore, find it advisable to reduce barriers to transition, while mental healthcare providers should be prepared to support transgender clients in seeking out, preparing for, and obtaining these services. Nevertheless, given the continued vulnerability of this population, mental healthcare practitioners should also be prepared to develop and reinforce this populations' resiliency against antitransgender stigma and discrimination, as well as prepare them for the possible increase of stress during transition. This may also include supporting and participating in policy and legislative measures arguing for transgender healthcare and human rights protections and decrying attempts to discriminate against this population (e.g., The American Psychiatric Association's *Position Statement on Transgender and Gender Variant Individuals*,^104^ The American Medical Associations' resolution on *Removing Financial Barriers to Care for Transgender Patients*,^105^ and the American Academy of Pediatrics *Statement in Opposition of Legislation that Discriminates Against Transgender Children*).^106^

### Limitations

The results of this meta-synthesis are very preliminary and there are a number of limitations inherent to a study of this kind, relying as it does on datasets that are methodologically varied. For example, relatively few studies reported on lifetime suicidality among individuals identified as GNC and CD, with the result that the analysis here is limited in generalizability. Similarly, relatively few studies measured suicidality either *before transition* or in the *past year* and it was, therefore, necessary to amalgamate several similar categories into the *before transition* group (*before treatment*, when *participants first felt trans*, and *before transition*). As noted, beyond the criteria set for an individual study's inclusion in this meta-synthesis and the actions taken to account for variation in their calculation of suicidality, no additional measures were taken to account for varying study designs and methodology.

Similarly, as is true within the broader field of transgender health, definitions of gender identity vary widely across these 42 studies. While documentation of nonbinary identities has increased in recent years^107^ researchers continue to struggle to account for the diversity among nonbinary individuals or respond to the rapid shifts in acceptable and preferred language within this community.^108^

The manner in which researchers inquired about suicidality is also concerning. For example, some researchers^63^ note that yes/no questions, such as “have you ever attempted suicide?” tend to overestimate positive responses from those who have self-harmed, but not attempted to end their life. This might be corrected by questioning participants' intent to die through in-person interviews, which have been found to reduce attempt rates from 4.6% to 2.7% of an adult sample.^109,110^ Unfortunately, only 14% of the 42 studies included here collected data primarily from face-to-face interviews.

### Future research

There is a lack of research into transgender people's experiences of minority stress^111^ and resiliency^61^ particularly the impact of transition-related interventions on suicidality.^52^ Researchers may also wish to include an additional measure reflecting current lived gender presentation which, owing to prejudice and/or other practical concerns, may differ from ones' core identity. It may also be the case that participants' gender identity evolves and shifts, and it is therefore difficult to capture this without asking participants to account for all previously held identities, rather than just that held in the moment of data collection.

Additionally, researchers might also explore the potential for bias in studies conducted in clinical environments, as participants may be dependent on the clinical environment for transition-related care. Consistent with social desirability bias, asking about suicidality in these environments, especially if not anonymously, may reinforce a *transitional narrative* where participants feel pressured to give a more positive picture of their mental health for fear of being denied, losing, or being forced to wait longer than necessary for life-saving transition-related services^112,113^ Indeed, the practice of delaying transitional treatment due to co-morbid symptomatology like depression and suicidality is widely practiced,^114^ though evidence is mounting against the efficacy and ethics of this approach.^78^ While set guidelines for transgender suicidality research would help to address this and other concerns, such a tool might not be help to address the expectation of bias created by the optics of the situation (e.g., that patients may not feel comfortable sharing their experiences of suicidality with those responsible for their care, where they are suspected to impact it).

Several decades of research have been conducted regarding suicidality experienced by transgender individuals. As summarized in the current meta-synthesis, rates of both ideation and attempts vary a great deal in subgroups of transgender individuals (FTM, MTF, GNC, CD), and based on several factors (e.g., type of questions asked, data collection method). The current meta-synthesis is the first of its kind to summarize 19 years of this important research and make sense of the 42 studies published in that time. It is our hope that the current article is a small but important addition to the body of knowledge regarding transgender suicidality and that the results may be used to inform future research and best practices.

## Abbreviations Used

CD: cross-dressers
FAAB: female assigned at birth
FTM: female-to-male
GNC: gender non-conforming
MAAB: male assigned at birth
MTF: male-to-female
